# Systematic identification of ACE2 expression modulators reveals cardiomyopathy as a risk factor for mortality in COVID-19 patients

**DOI:** 10.1186/s13059-021-02589-4

**Published:** 2022-01-10

**Authors:** Navchetan Kaur, Boris Oskotsky, Atul J. Butte, Zicheng Hu

**Affiliations:** 1 Bakar Computational Health Sciences Institute, University of California, San Francisco, San Francisco, CA USA; 2 Department of Pediatrics, University of California, San Francisco, CA USA; 3 Department of Microbiology and Immunology, University of California, San Francisco, San Francisco, CA USA

## Abstract

**Background:**

Angiotensin-converting enzyme 2 (ACE2) is the cell-entry receptor for SARS-CoV-2. It plays critical roles in both the transmission and the pathogenesis of COVID-19. Comprehensive profiling of ACE2 expression patterns could reveal risk factors of severe COVID-19 illness. While the expression of ACE2 in healthy human tissues has been well characterized, it is not known which diseases and drugs might be associated with ACE2 expression.

**Results:**

We develop GENEVA (GENe Expression Variance Analysis), a semi-automated framework for exploring massive amounts of RNA-seq datasets. We apply GENEVA to 286,650 publicly available RNA-seq samples to identify any previously studied experimental conditions that could be directly or indirectly associated with ACE2 expression. We identify multiple drugs, genetic perturbations, and diseases that are associated with the expression of ACE2, including cardiomyopathy, HNF1A overexpression, and drug treatments with RAD140 and itraconazole. Our joint analysis of seven datasets confirms ACE2 upregulation in all cardiomyopathy categories. Using electronic health records data from 3936 COVID-19 patients, we demonstrate that patients with pre-existing cardiomyopathy have an increased mortality risk than age-matched patients with other cardiovascular conditions. GENEVA is applicable to any genes of interest and is freely accessible at http://genevatool.org.

**Conclusions:**

This study identifies multiple diseases and drugs that are associated with the expression of ACE2. The effect of these conditions should be carefully studied in COVID-19 patients. In particular, our analysis identifies cardiomyopathy patients as a high-risk group, with increased ACE2 expression in the heart and increased mortality after SARS-COV-2 infection.

**Supplementary Information:**

The online version contains supplementary material available at 10.1186/s13059-021-02589-4.

## Background

Coronavirus disease 2019 (COVID-19) is an infectious disease caused by severe acute respiratory syndrome coronavirus 2 (SARS-COV-2). The World Health Organization (WHO) declared the COVID-19 outbreak a pandemic on March 11, 2020. As of August 10, 2021, there have been 200 million recorded COVID-19 cases and over 4 million deaths [1].

Angiotensin-converting enzyme 2 (ACE2) is the cell-entry receptor for SARS-CoV-2 [2]. The binding between ACE2 and spike (S) protein of SARS-COV-2 initiates the viral entry into target cells. ACE2 plays key roles in both the transmission and pathogenesis of SARS-CoV-2, as demonstrated by the following lines of evidence: (1) SARS-CoV-2 fails to infect the lung-derived cell line A549 in the absence of ACE2 expression. The infection is restored after overexpressing ACE2 in the cell line [3]. (2) SARS-CoV-2 fails to infect wild-type mice but can infect and cause pneumonia in transgenic mice expressing human ACE2 [4, 5]. (3) COVID19-related tissue damages are detected in organs with ACE2 expression, including lungs, intestines, colons, and hearts [6–8]. (4) ACE2 expression is increased in the lungs of patients with comorbidities associated with severe COVID-19, suggesting that the level of ACE2 expression is associated with disease severity [9]. Taking these lines of evidence together, it is crucial to comprehensively characterize the ACE2 expression in human tissues.

To comprehensively profile the expression patterns of ACE2, we not only need to characterize its expression in healthy tissues but also identify diseases, drugs, and genetic perturbations that are associated with ACE2 expression changes. The expression of ACE2 in healthy human tissues has been well characterized by resources such as the Human Cell Atlas and GTEx, with the highest expression detected in the intestine, testis, lung, cornea, heart, kidney, and adipose tissues [10, 11]. However, it is still not clear which diseases and drugs are associated with the ACE2 expression. Since ACE2 expression is tightly linked with the pathogenicity of SARS-COV-2, characterizing the expression pattern of ACE2 in different conditions will help us reveal and explain the risk factors of severe illness from COVID-19.

RNA-sequencing data profiles the full transcriptome of samples. Currently, more than 200,000 human RNA-seq samples are publicly available, providing an unprecedented opportunity for us to examine ACE2 expression in different human cell types under a variety of conditions and treatments. Data harmonization efforts such as ARCHS4 have uniformly preprocessed the RNA-seq data, making them readily available for analysis [12]. However, fully automated analysis of these datasets faces two main obstacles. First, the metadata are non-standardized and are often unstructured, making it difficult to extract experimental conditions from the studies. Second, experimental designs are highly variable. While some studies adopt the simple control-versus-treatment design, other studies are more complicated, involving multiple time points, combination treatments, or stratified cohorts. The heterogeneous design makes it difficult to analyze the datasets using a single statistical model.

Multiple tools have been made to analyze transcriptomics data, including CREEDS [13], scanGEO [14], GEM-TREND [15], StarGEO [16], SIGNATURE [17], SPIED [18], Cell Montage [19], ProfileChaser [20], ExpressionBlast [21], and SEEK [22]. However, the existing tools have several limitations, preventing them from fully exploring the publicly available RNA-sequencing resources. First, some of the tools annotate the metadata manually and are unable to cover the large number of datasets currently available. Second, the tools focus on differential expression analysis between two groups (e.g., control versus treatment), preventing them from analyzing studies with more complex study designs.

In this study, we developed GENEVA (GENe Expression Variance Analysis), a semi-automated framework for exploring public RNA-seq datasets. For a given gene, GENEVA identifies the most relevant datasets by analyzing the variance of the gene expression. GENEVA visualizes the relevant datasets for detailed manual analysis. GENEVA is scalable and is agnostic to study designs. Using GENEVA, we identified multiple drugs, genetic perturbations, and diseases that modulate the expression of ACE2, including cardiomyopathy, HNF1A overexpression, and drug treatments with RAD140 and itraconazole. Our in-depth meta-analysis of seven datasets reveals increased ACE2 expression in all cardiomyopathy categories. By analyzing the clinical data of 3936 COVID19 patients at UCSF hospital, we demonstrate that patients with pre-existing cardiomyopathy have an increased mortality risk than other patients, including propensity score-matched patients with other cardiovascular conditions.

## Results

### Analysis of 286,650 RNA-seq samples reveals complex transcriptional networks of ACE2.

Our study leverages human RNA-sequencing data from the ARCHS4 project, containing 286,650 uniformly preprocessed data from 9124 Gene Expression Omnibus (GEO) series [12]. The large number of RNA-sequencing samples provides an unprecedented resource for studying the expression of ACE2 in different human cell types under a variety of conditions and treatments.

We first characterized the transcriptional networks of ACE2 using all 286,650 samples. We calculated the Pearson correlation between ACE2 and all other human genes. Because of the large sample size, most of the correlations are statistically significant, even after multiple testing adjustments. Therefore, we focused on the correlation coefficients themselves as a measure of effect size, rather than the *p* values or significance. While most of the genes have correlation coefficients near 0, a small set of genes are highly correlated with ACE2, with the highest correlation to be 0.72 between FABP2 and ACE2 (Additional file 1, Fig. S1A and B). The top correlated genes include FABP2, MEP1B, and transcription factors such as HNF4G (Top 30 genes shown in Additional file 1, Fig. S1B-C, and all correlations listed in Additional file 2 Table S1 and Additional file 3 Table S2). The top correlated pathways include multiple pathways related to the digestive process (Top 30 pathways shown in Additional file 1, Fig. S1D, and all pathways listed in Additional file 4, Table S3), consistent with the high expression of ACE2 in small intestines.

Dataset from the GEO database covers a variety of tissue types. To see how the tissue differences affect the correlation between ACE2 and other genes, we examined tissue-specific transcriptome data from the GTEx consortium. We found similar co-expression relationship between ACE2 and other genes using data from GTEx or GEO (correlation = 0.42, *p* value < 0.0001, Additional file 5, Table S4), suggesting that the co-expression profile between ACE2 and other genes are heavily influenced by the tissue differences. To avoid our analysis being dominated by tissue differences, we evaluated the correlations between ACE2 and other human genes within each RNA-seq dataset. We then calculated the mean and variances of the correlations across all datasets. Our analysis reveals a positive relationship between the mean and variance of the correlation coefficients (Additional file 1, Fig. S1E). While some genes have a high average correlation with ACE2, their correlation with ACE2 is highly variable in individual datasets. The results suggest against a common transcriptional network around the ACE2 gene. Rather, ACE2 is co-regulated with different sets of genes under different conditions.

We adopted a mixed-effect model to estimate the overall association between ACE2 and other genes across studies, allowing us to prioritize genes with relatively conserved correlations with ACE2 across all studies. The top genes include MYO7B, CALML4, and transcription factors such as HNF1A (Top 30 genes shown in Additional file 1, Fig. S1 F-G, and all genes listed in Additional file 6, Table S5 and Additional file 7, Table S6). Pathway analysis identified many metabolic processes to be correlated with ACE2 expression. The findings are consistent with previous observations that ACE2 is involved in glucose metabolism and energy stress responses (Top 30 pathways shown in Additional file1 Fig. S1 H, and all pathways listed in Additional file 8, Table S7) [23, 24].

Our analysis identified three transcription factors in the hepatocyte nuclear factor family, including HNF4G, HNF1A, and HNF4A (Additional file 1, Fig. S1 C and G). HNF4G has the highest overall correlation with ACE2 while HNF1A has the highest standardized correlation coefficient within studies. We then tested the causal relationship between the transcription factors and ACE2 expression. We identified two RNA-seq datasets that compared human cells with or without genetic perturbation of HNF4G and HNF1A. While HNF4G is positively correlated with ACE2 expression (Additional file 1, Fig. S1 C), overexpression of HNF4G does not lead to significantly increased ACE2 expression (Additional file 1, Fig. S1I). Rather, there is a trend of reduction in ACE2 expression. HNF1A overexpression leads to increased ACE2 expression and HNF1A knockdown reduced ACE2 expression (Additional file 1, Fig. S1J) in LNCaP cells, a prostate cancer cell line. The result is consistent with the positive correlation between HNF1A and ACE2 (Additional file 1, Fig. S1G). A previous study showed that HNF1A induces ACE2 in pancreatic islets [25]. Our result in a prostate cancer cell line further confirmed the role of HNF1A in regulating ACE2. However, it should be noted that HNF1A and ACE2 are not correlated in all RNA-seq datasets (Additional file 1, Fig. S1K), demonstrating the complexity of ACE2 regulation in different tissues.

### Gene expression variance analysis reveals diseases and therapeutics that modulate the expression of ACE2

**Fig. 1:**
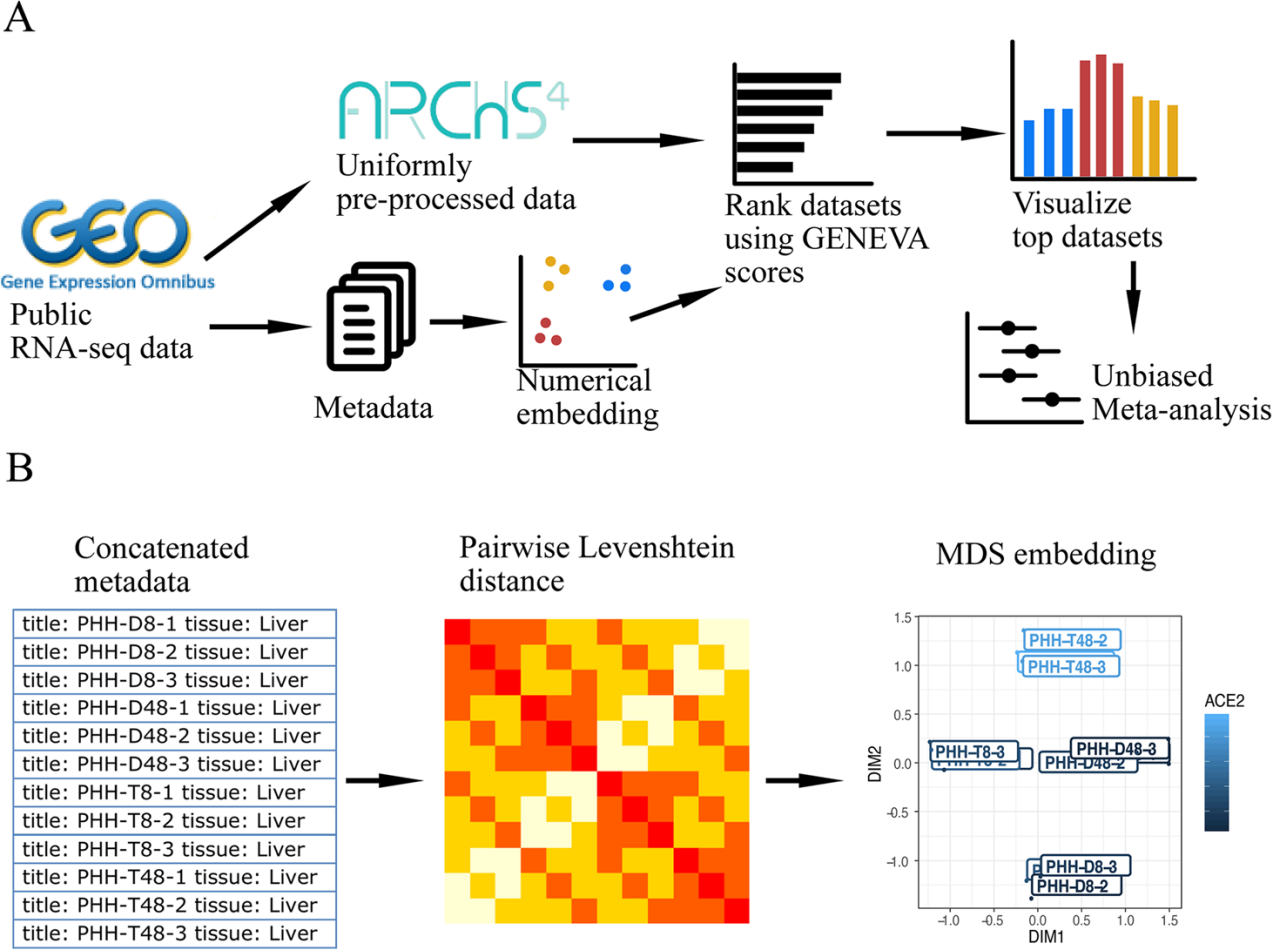
Mining RNA-seq data using Gene Expression Variance Analysis (GENEVA). **A** The workflow of GENEVA. **B** The procedure for embedding metadata into numerical vectors

Next, we hope to identify conditions that are associated with the expression of ACE2. We developed a computational framework named GENEVA (Gene Expression Variance Analysis) to identify the most relevant datasets for visualization and detailed manual analysis (Fig. 1A and “Methods”). GENEVA prioritizes the datasets that have a large variance of ACE2 expression. The rationale is that datasets with large ACE2 variance are likely to contain conditions that modulate the ACE2 expression. At the same time, GENEVA controls for the overall heterogeneity of the samples to prioritize datasets in which ACE2 is specifically modulated by experimental conditions rather than due to tissue type differences. In addition, GENEVA embeds the metadata into numerical space and prioritizes datasets with high correlations between ACE2 expression and the metadata (Fig. 1B). This allows GENEVA to identify datasets in which ACE2 is regulated by experimental conditions rather than randomness or unexplained factors. While our study focuses on ACE2 and its role in COVID-19 disease, GENEVA is applicable to all genes. We created a web application that allows researchers to apply GENEVA to their gene of interest [http://genevatool.org].

We tested the significance of the GENEVA scores using a permutation procedure. We randomly shuffle the samples across studies to generate a null distribution. We compared each GENEVA score to the null distribution to calculate the *p* value. We adjusted for multiple testing using the false discovery rate (FDR) method [26]. We identified 27 significant datasets with FDRs less than 0.05 (Table 1). Interestingly, GENEVA identified HNF1A as an ACE2 modulator, which was also identified in our correlation analysis (Additional file 1, Fig. S1J). GENEVA additionally identified multiple drugs and diseases that modulate or are associated with the ACE2 expression, revealing potential risk factors for severe illness from COVID-19.

**Table 1:**
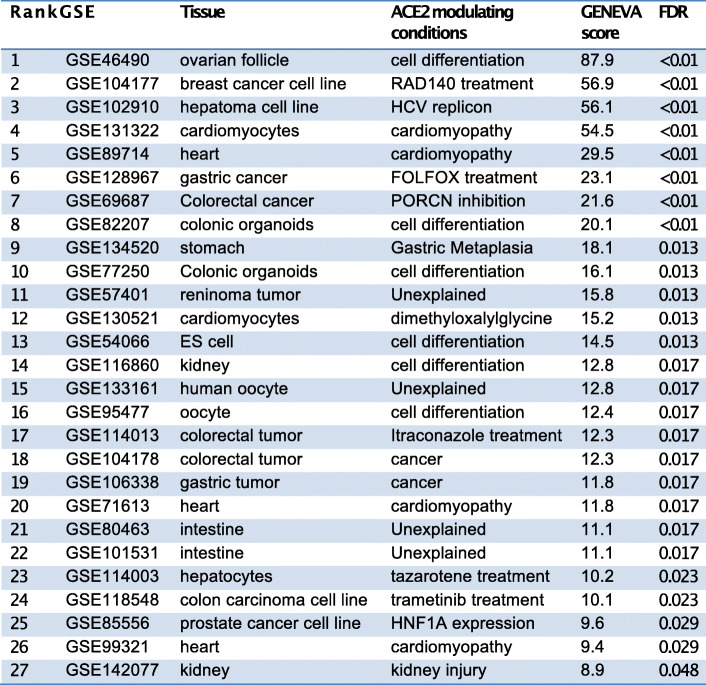
RNA-seq datasets with GENEVA scores of ACE2 expression that are statistically significant

**Fig. 2:**
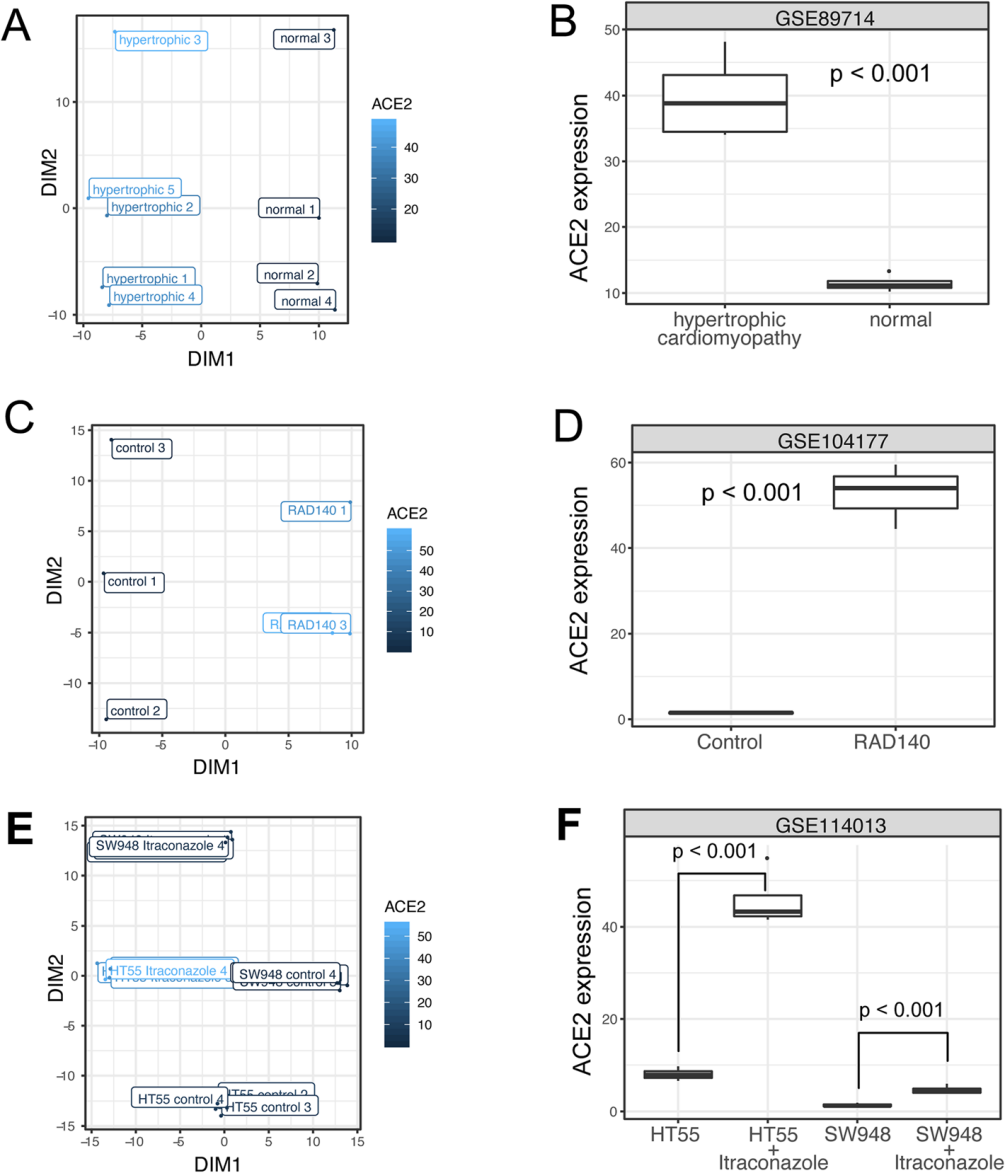
Highlighted conditions that modulate ACE2 expression. **A,B** Plots showing data from a study with GEO accession GSE89714. **A** A scatter plot showing the association between ACE2 expression and the embedded metadata. All metadata fields are concatenated for embedding. The plot only shows the sample title as labels. The labels are jittered to avoid perfect overlap. The color code represents the ACE2 expression level. **B** Box plot showing the ACE2 expression in normal hearts and hearts with hypertrophic cardiomyopathy. **C,D** Plots visualizing data from a study with GEO accession GSE104177, showing the ACE2 expression in breast cancer xenografts with or without RAD140 treatment. **E,F** Plots visualizing data from a study with GEO accession GSE114013, showing the ACE2 expression in two prostate cancer cell lines with or without itraconazole treatment

Here, we highlight three ACE2-modulating conditions, manually picked based on their effect on ACE2 expression and their potential impact on public health. Data from GSE89714 show upregulated expression of ACE2 in hypertrophic cardiomyopathy (Fig. 2A, B). Hypertrophic cardiomyopathy is the most common inherited heart disease, affecting an estimated 15,188,000 individuals (0.2%) worldwide [27]. Our finding is consistent with an increased death rate in COVID-19 patients with heart conditions [28–30] and suggests that higher ACE2 expression can contribute to the increased risk. Data from GSE104177 showed that RAD140, a selective androgen receptor modulator, induces ACE2 expression in human breast cancer xenografts (Fig. 2C, D). Data from GSE114013 show that itraconazole, an antifungal drug, upregulates ACE2 expression in two colorectal cancer cell lines, HT55 and SW948 (Fig. 2 E, F). These findings suggest that these drugs should be studied with respect to ACE2 expression in lung and cardiac cells and tissues and that patients on these drugs should be studied closely during the pandemic. If these subsequent studies do continue to suggest this effect on increasing ACE2 expression, heightened caution could be warranted when using these drugs during the COVID-19 pandemic.

### Joint analysis shows ACE2 upregulation in all types of cardiomyopathy

GENEVA prioritizes datasets with large variances in ACE2 expression. However, the procedure may introduce bias, as studies with small ACE2 variations are ignored. Consider an example in which multiple studies have profiled the effect of a drug. Some studies show that the drug upregulates ACE2 while other studies show that the drug has no effect on ACE2. The effect of the drug will be overestimated if a researcher only includes the studies with positive results. Therefore, after the GENEVA analysis, a joint analysis of all related datasets is required to confirm the findings.

We performed a comprehensive search for datasets related to the three highlighted conditions, including cardiomyopathy, itraconazole treatment, and RAD140 treatment. We did not find additional datasets related to itraconazole and RAD140 treatment. For cardiomyopathy, we identified a total of 7 datasets. We performed a meta-analysis using a mixed-effect model (with cardiomyopathy as the fixed effect and the dataset as the random effect), taking data from all 7 datasets into account. The result confirmed that ACE2 expression is significantly elevated in heart tissue samples from cardiomyopathy patients (*p* value < 0.001).

**Fig. 3:**
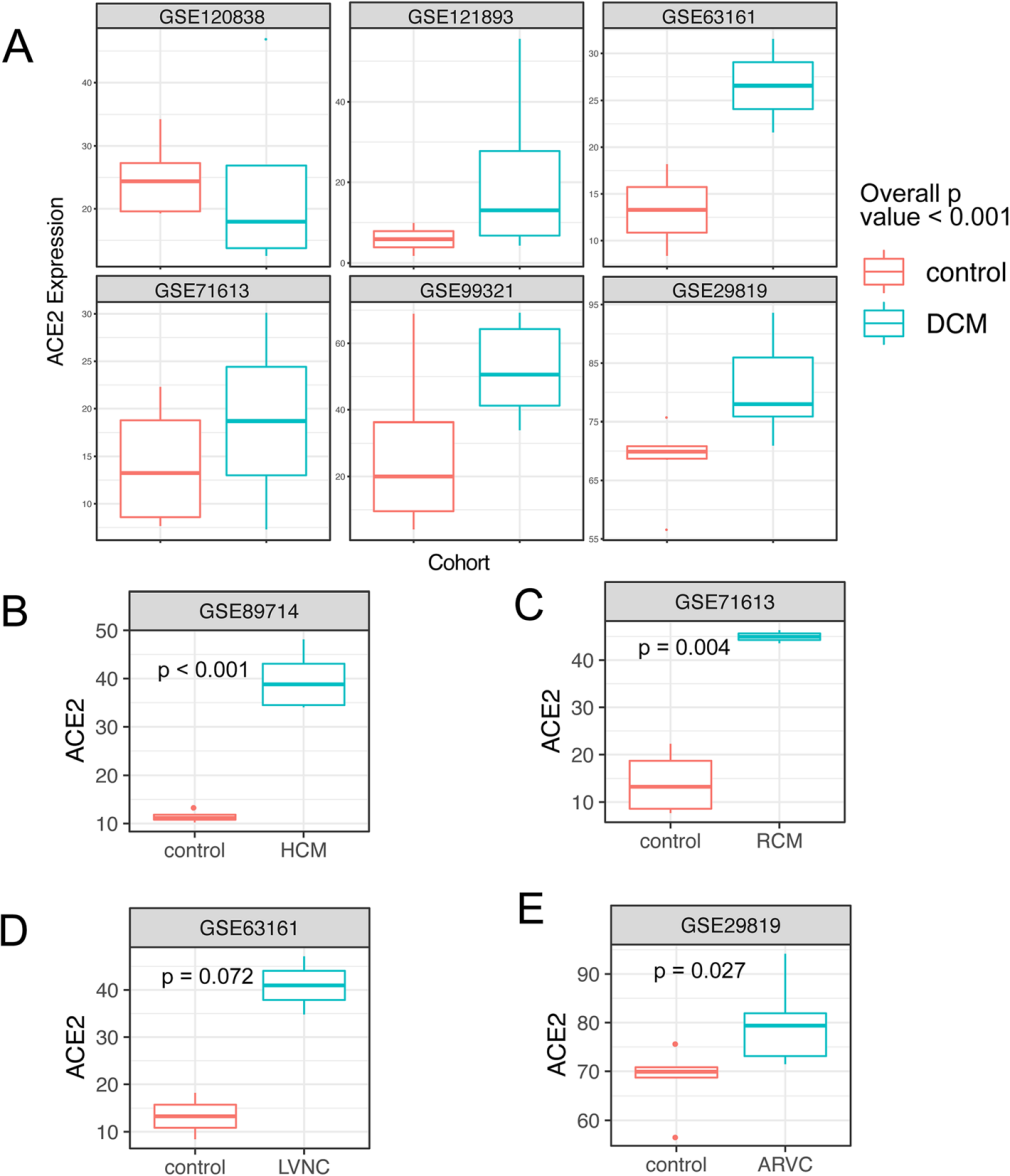
Meta-analysis confirms ACE2 upregulation in all major types of cardiomyopathy. **A** The ACE2 expression in normal hearts and hearts with DCM. Data are from five RNA-seq datasets and one microarray dataset. The overall *p* value is calculated using a mixed model, with the dataset as the random effect and DCM as the fixed effect. **B–E** The ACE2 expression in normal hearts and hearts with HCM, RCM, LVNC, and ARVC. *p* values in **B–E** are calculated using *t*-tests

We next examined the ACE2 expression in different types of cardiomyopathy. The most common types of cardiomyopathies include dilated cardiomyopathy (DCM), hypertrophic cardiomyopathy (HCM), restrictive cardiomyopathy (RCM), arrhythmogenic right ventricular cardiomyopathy (ARVC), and left ventricular noncompaction (LVNC) [31–35]. These cardiomyopathy types have different causes and show distinctive morphology and physiology characteristics. Although previous studies have demonstrated ACE2 upregulation in DCM and HCM [36, 37], how ACE2 is regulated in other types is unknown. Within the 7 datasets, we were able to identify all the common cardiomyopathy types. Our analysis revealed significantly increased ACE2 expression in most of the cardiomyopathy types (Fig. 3), including DCM, HCM, RCM, and LVNC. Although the result of ARVC is not statistically significant, the data show a clear trend of ACE2 upregulation (Fig. 3D). We performed Egger regression and did not observe significant publication bias in the cardiomyopathy datasets [38] (Additional file 1, Figure S2).

### COVID-19 patients with pre-existing cardiomyopathy show an increased mortality rate

While COVID19 patients with cardiovascular conditions show a higher mortality rate, it is not clear how cardiomyopathy, in particular, affects the survival of the patients. Because the ACE2 expression is significantly elevated in the heart of cardiomyopathy patients, we hypothesize that pre-existing cardiomyopathy leads to increased mortality in patients with COVID19.

We identified 3936 COVID19 patients from the electronic health records (EHR) of the University of California San Francisco (UCSF) hospital. We divided the patients into three groups, including patients with pre-existing cardiomyopathy (*N* = 43), patients with other pre-existing cardiovascular diseases (*N* = 624), and patients without cardiovascular diseases (*N* = 3269) (Table 2). The most common non-cardiomyopathy cardiovascular diseases include hypertension (*N* = 424), atherosclerotic heart diseases (*N* = 120), and cardiac arrhythmia (*N* = 105).

**Table 2:**
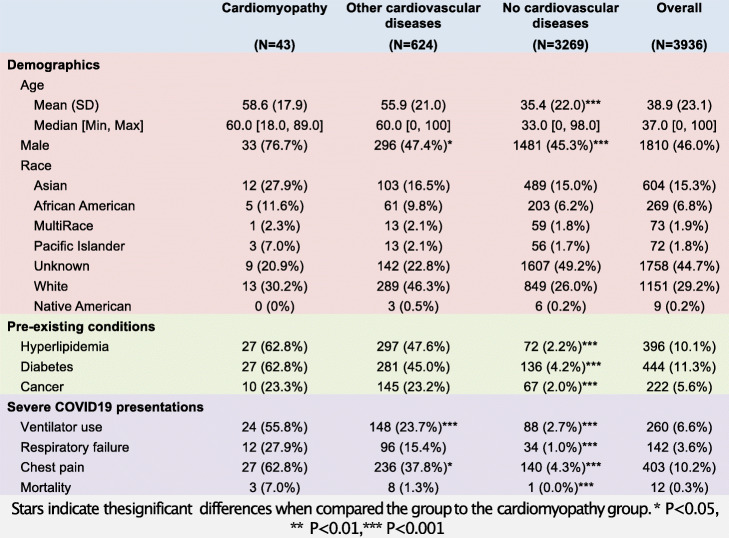
Demographic and clinical information of COVID-19 patients

**Fig. 4:**
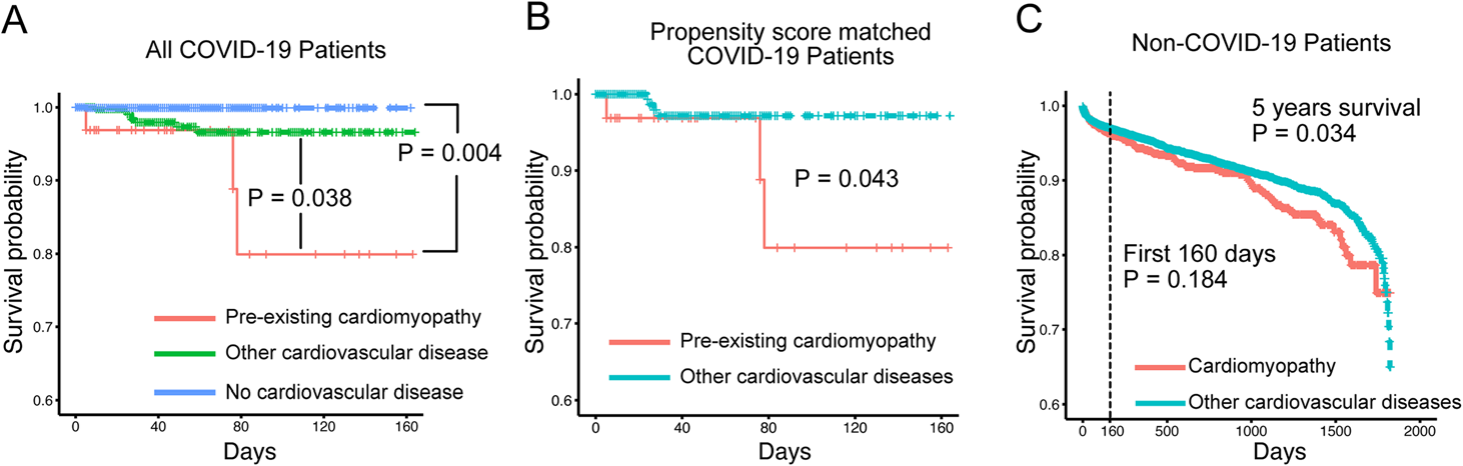
Patients with pre-existing cardiomyopathy show an increased mortality rate. **A** Kaplan-Meier curve of COVID19 patients with pre-existing cardiomyopathy (*N* = 43), patients with other pre-existing cardiovascular diseases (*N* = 624), and patients without cardiovascular diseases (*N* = 3269). *P* values are from Cox proportional-hazards models, controlling for differences in the demographics and non-cardiovascular conditions between the groups. **B** Kaplan-Meier curve of COVID19 patients with pre-existing cardiomyopathy (*N* = 43) and a cohort of propensity score-matched patients with other pre-existing cardiovascular diseases (*N* = 344). *P* values are from a log-rank test. **C** Kaplan-Meier curve of COVID-19-negative patients with pre-existing cardiomyopathy (*N* = 2250) and a cohort of propensity score-matched patients with other pre-existing cardiovascular diseases (*N* = 18,000). *P* values are from a log-rank test

We first compared the cardiomyopathy patients to patients without cardiovascular diseases. Patients with cardiomyopathy have a larger proportion of males and older ages. They also have a higher percentage of patients with pre-existing conditions such as cancer, diabetes, and hyperlipidemia. A higher percentage of cardiomyopathy patients have severe COVID-19 disease presentations, including ventilator use, respiratory failure, chest pain, and death (Table 2). We then performed survival analysis to test the effect of cardiomyopathy while controlling for differences in age, gender, and pre-existing conditions using a multivariable Cox proportional-hazards model. We confirmed that cardiomyopathy is significantly associated with the risk of death (*p* = 0.004) (Fig. 4A).

We next compared the cardiomyopathy patients to patients with other cardiovascular diseases. Cardiomyopathy patients have a higher proportion of males compared to patients with other cardiovascular diseases. Age, race, and pre-existing conditions are comparable between the two groups. Again, we observe that a higher percentage of cardiomyopathy patients have severe COVID-19 presentations, including ventilator use, chest pain, and death (Table 2). Multivariable Cox proportional-hazards regression confirms that cardiomyopathy is significantly associated with the risk of death (*p* = 0.038, 438% increase in observed death rate) (Fig. 4A). We further confirmed the increased mortality by comparing cardiomyopathy patients with a propensity score-matched cohort of patients with other cardiovascular diseases (Fig. 4B and Additional file 1, Table S8).

We then examined the survival of cardiomyopathy patients who are COVID-19 negative. We compared the survival of COVID-19-negative cardiomyopathy patients (*N* = 2250) with a propensity score-matched cohort of patients with other cardiovascular diseases (*N* = 18,000). The two cohorts are comparable in demographics and non-cardiovascular diseases (Additional file 1, Table S9). The 5-year mortality rate is only slightly higher in the cardiomyopathy patients (*p* = 0.034, 22% increase in observed death rate) (Fig. 4C). When we consider the patient’s survival at 160 days, a time frame comparable to the COVID-19-positive dataset, there is no significant difference between the survival of the two groups (Fig. 4C).

Taken together, the results show that cardiomyopathy itself does not pose large additional risk of mortality among patients with cardiovascular diseases. Rather, the interaction between SARS-CoV-2 infection and pre-existing cardiomyopathy leads to increased mortality in patients. Our transcriptomics analysis suggests that the upregulated ACE2 expression may contribute to the disease severity of COVID-19 in patients with pre-existing cardiomyopathy. However, further mechanistic studies are needed to establish the causal relationship between ACE2 upregulation and mortality in COVID-19 patients.

## Discussion

The disease severity of COVID-19 patients varies from asymptomatic to life-threatening. While we do not fully understand the reason behind such variation, it is clear that the disease severity is determined by multiple factors, including age, gender, the status of the immune system, and the pre-existing conditions [7, 39–41]. ACE2 expression is a key determinant of the disease severity, as shown by multiple studies in humans and in animal models [3, 5, 9]. Therefore, it is critical to identify conditions are associated ACE2 expression, as the information will help us reveal and explain factors associated with increased risk of severe illness from COVID-19.

We leveraged the massive amount of publicly available RNA-seq data to identify the ACE2-modulating conditions. While many tools exist for analyzing bulk GEO data, they are not optimized for this purpose. First, some tools require researchers to search for datasets using keywords, such as the name of a drug or a disease. These tools do not address our needs, as we are looking for any conditions that modulate ACE2 expression. Second, some tools require manual annotation of experimental groups in the studies, which are not scalable and often only cover a small subset of currently available datasets. Finally, the existing tools focus on differential expression analysis of two groups and are unable to address more complex experimental designs.

We address the problems using a variance analysis approach. Instead of comparing two experimental groups, we quantify the variance of the gene expression across all samples in a study. The rationale is that datasets with large ACE2 variances are likely to contain conditions that are associated with ACE2 expression. We improved our approach by using two modifications. First, we numerically embedded the metadata and calculated the regression coefficient *R*^2^ between ACE2 and the embedding. This allows us to prioritize datasets in which the ACE2 variation is associated with metadata. Second, we controlled for the overall heterogeneity of samples in the study, this allows us to prioritize datasets in which ACE2 are specifically modulated rather than as a result of cell-type differences.

Our study identifies multiple diseases, conditions, and genetic perturbations that are associated with ACE2 expression. When interpreting these findings, readers should take into account several limitations of our study. First, many of the conditions are discovered based on data from one study with small sample sizes. These results should be viewed as data-driven hypotheses rather than definitive proofs. Additional data is required to confirm the findings. Second, our analysis does not establish a direct link between COVID-19 risk and the identified ACE2 modulators. Clinical studies are required to test if these ACE2-modulating conditions alter the risk of COVID-19 infection and pathogenesis. Third, many of the RNA-seq datasets are derived from observational studies. The association between conditions and ACE2 expression does not indicate causal relationships. Finally, the datasets profile the ACE2 expression in bulk tissues. Therefore, it is not clear if the variation of ACE2 expression is due to a change in cellular composition or a change in transcriptional regulation. Single-cell analysis is required to identify the cause of ACE2 expression change.

Heart diseases are associated with severe COVID-19 illness through two mechanisms. Pre-existing heart conditions are comorbidities of COVID19 [28, 41]. On the other hand, SARS-COV-2 infection can induce acute myocardial injury [30]. Cardiomyopathy is one of the most common heart diseases. Compared to other age-related heart conditions, cardiomyopathy can affect individuals at any age. However, clinical studies were lacking to specifically characterize the disease severity of COVID-19 in patients with cardiomyopathy. Our transcriptional analysis highlights the significantly increased ACE2 expression in the hearts of cardiomyopathy patients, suggesting that cardiomyopathy patients are at an increased risk of heart damage caused by COVID-19.

Consistent with the RNA-seq finding, we found that COVID19 patients with pre-existing cardiomyopathy show increased mortality risk than other populations, even compared with age-matched populations with other cardiovascular conditions. While mechanistic research is needed to establish the causal relationship between ACE2 upregulation and the increased mortality, our result identifies the cardiomyopathy patients as a high-risk group that needs extra protection and care.

This project demonstrated public RNA-sequencing data as a valuable resource for biomedical knowledge discovery. The existing RNA-seq data can be quickly repurposed to address pressing problems, such as identifying the risk factor of COVID19. While this study focused on analyzing the expression of ACE2, researchers can identify modulators for any genes or gene signatures of interest using the GENEVA web portal at genevatool.org.

## Conclusions

We applied GENEVA (GENe Expression Variance Analysis) to 28,6650 publicly available RNA-seq samples to identify any previously studied experimental conditions that could directly or indirectly modulate ACE2 expression. We identified multiple drugs, genetic perturbations, and diseases that modulate the expression of ACE2, including cardiomyopathy, HNF1A overexpression, and drug treatments with RAD140 and itraconazole. Our unbiased meta-analysis of seven datasets confirms ACE2 upregulation in all cardiomyopathy categories. Using electronic health records data from 3936 COVID19 patients, we demonstrate that patients with pre-existing cardiomyopathy have an increased mortality risk than matched patients with other cardiovascular conditions.

## Methods

### Data preparation

We downloaded the uniformly processed RNA-seq data from ARCHS4 website (https://amp.pharm.mssm.edu/archs4/download.html) on August 03, 2020. The downloaded data include gene-level count data of 286,650 samples from 9124 datasets and sample-level metadata. We transformed the gene count data into percentile rank data, which reduces the influences of library size, batch effects, and extreme values [42, 43]. We downloaded study-level metadata using the entrez_search and entrez_summary function from the rentrez library [44].

### Co-expression analysis

We first calculated the Pearson correlation between ACE2 and other genes using data from all 286,650 samples. For related dataset that share samples with each other, we only included one dataset with the largest sample size and exclude the other related dataset to make sure the studies are independent. Transcription factors are identified by selecting genes with the Gene Ontology term “DNA-binding transcription factor activity (GO:0003700).” We also performed mixed-effect regression to assess the association between ACE2 and other genes while controlling for study differences [Gene ~ ACE2 (fixed effect) + dataset ID (random effect for intercept and slope)]. The lme4 R package was used to fit the mixed-effect models. To identify pathways associated with ACE2, we used the associations with ACE2 (measured by the *t* statistics of the fixed effect in the mixed-effect model) as a signature. We used the signature to query the Gene Ontology Biological Process database [45]. The fgsea function from the fgsea library was used to calculate the enrichment score [46].

### Metadata embedding

We first concatenated the metadata of each sample into a single string, including the title, tissue type, and other characteristics (e.g. demographics, time points, treatment, genetic information, and disease status). We then calculated the pairwise Levenshtein distance between the strings that belong to the same study (GEO series). We applied multidimensional scaling to the pairwise Levenshtein distance and embedded the strings into 2-dimensional space for visualization and downstream analysis.

### GENEVA analysis

For a given gene in a given dataset, we first calculated the variance of the gene (VARg). We measure the overall heterogeneity of the samples by calculating the average variance of all genes (VARm). We run a regression using the expression of the gene as the dependent variable and the embedded metadata as independent variables (expression ~ first embed dimension + second embed dimension). The regression coefficient (*R*^2^) represents the association between the expression of the gene and the embedded metadata. The product between VARg and *R*^2^ represents the variance of the gene explained by the embedded metadata. The GENEVA score is defined as VARg × *R*^2^ / VARm.

To test the significance of the GENEVA scores, we shuffled the samples within each dataset. We then calculated the GENEVA scores of all shuffled datasets to create a null distribution. Given a GENEVA score G, its *p* value is defined as the probability that the null distribution is greater than G: *p* value = Prob(null > G). We adjust the *p* values for multiple testing using the false discovery rate method.

### Joint analysis of cardiomyopathy datasets

We searched the gene expression omnibus using the keyword “cardiomyopathy.” We then filter the results to only include studies that (1) profiled the transcriptome of heart tissues from humans and (2) compared cardiomyopathy samples with healthy samples. We identified 7 studies. We used a mixed-effect model to test the effect of cardiomyopathy on ACE2 expression: ACE2 expression ~ study (random effect) + cardiomyopathy status (fixed effect).

To examine the ACE2 expression in different types of cardiomyopathy, we separated the cardiomyopathy samples based on their subtype. We matched the cardiomyopathy samples with healthy controls within the same study. We used unpaired *T*-tests to test the effect of each cardiomyopathy type on ACE2 expression. Since data from multiple studies are available for dilated cardiomyopathy (DCM), we used a mixed-effect model to test the effect of DCM on ACE2 expression: ACE2 expression ~ study (random effect) + cardiomyopathy status (fixed effect).

To test for publication bias, Egger regression was performed by fitting a linear model using effect size/standard error as the dependent variable and 1/standard error as the dependent variable. The *p* value of the intercept term was used to assess the significance of the publication bias.

### Analysis of electronic health records

The UCSF COVID-19 Data Mart records the clinical information of COVID19 patients and selected control patients using the Observational Medical Outcomes Partnership (OMOP) data format. We identified COVID19 patients from the clinical data using the ICD10 code U07. 1. We identified cardiomyopathy patients using the ICD10 codes I42 and I43, entered before their first COVID19 diagnosis. We identified patients with other cardiovascular diseases using the following ICD10 codes I00 - I99. We compared the cardiomyopathy patient with patients with other cardiovascular diseases and patients without cardiovascular diseases. We tested if the demographic and clinical variables are significantly different between the groups using single variable logistic regressions (cardiomyopathy ~ clinical variable). We used the coxph function in the survival R package to perform the survival analysis, controlling for the variables that are significantly different in the logistic regressions. Patient survival time is defined as the time between their first COVID19 diagnosis and their death date. Live patients are censored on the last day of their encounter. In the COVID-19-negative cohort, cardiomyopathy patients are defined as patients whose first cardiovascular-related diagnosis is cardiomyopathy (ICD10 code I42 or I43). Patients with other cardiovascular diseases are defined as patients who have cardiovascular diseases (ICD10 code I00 - I99), but do not have cardiomyopathy. Patient survival time is defined as the time between their first cardiovascular disease and their death. Live patients are censored on the last day of their encounter. To perform propensity score matching, we first calculated the propensity score using logistic regression (cardiomyopathy ~ age + race + gender + non-cardiovascular pre-existing conditions). We subsampled the non-cardiomyopathy cohort so that the distribution of its propensity score matches with the cardiomyopathy cohort.

### GENEVA web portal

GENEVA web tool is an open-source application available under GNU General Public License at http://genevatool.org. It is implemented in python web framework Django. The source code for the tool is available in the public Git repository at https://github.com/NavchetanKaur/geneva-webtool. The tool offers an intuitive interface and user guide on the home page. Users can select either of the two options from “Gene Query” and “Gene Signature Query” and query their gene of interest or set of upregulated and downregulated genes of interest. The results are displayed in tabular form with calculated GENEVA scores. GSE descriptions are further represented in plots and tables.

All experimental methods comply with the Helsinki Declaration.

## Supplementary information

**Additional file 1: Supplemental figures and tables.** This file including figure S1, figure S2, table S8 and table S9.

**Additional file 2: Table S1.** The correlation Pearson between ACE2 and other genes across all datasets.

**Additional file 3: Table S2.** The correlation Pearson between ACE2 and transcription factors across all datasets.

**Additional file 4: Table S3.** The enriched pathways related to genes that are correlated with ACE2 across all datasets.

**Additional file 5: Table S4.** Comparing the co-expression relationships of ACE2 in GEO and GTEx datasets.

**Additional file 6: Table S5.** The association between ACE2 and other genes, measured by the t statistics from mix-effect models that controls for study differences.

**Additional file 7: Table S6.** The association between ACE2 and transcription factors, measured by the t statistics from mix-effect models that controls for study differences.

**Additional file 8: Table S7.** The enriched pathways related to genes that are associated with ACE2, measured by the t statistics from mix-effect models that controls for study differences.

**Additional file 9:.** Review history.

## Data Availability

GENEVA web tool is an open-source application available under GNU General Public License at http://genevatool.org. The source code for the GENEVA analysis is in the public Git repository at https://github.com/NavchetanKaur/geneva-webtool. A stable version of the source code is deposited at Zenodo [47]. The uniformly processed RNA-seq data are available to be explored using the GENEVA webtool and can also be downloaded through the ARCHS4 website (https://amp.pharm.mssm.edu/archs4/download.html), including the datasets that are highlighted in the article (GSE89714 [48], GSE104177 [49], GSE114013 [50], GSE120838 [51], GSE121893 [52], GSE63161 [53], GSE71613 [54], GSE99321 [55], and GSE29819 [56]). The clinical data of COVID-19 from UCSF electronic health records is not shared at subject level due to institutional restrictions. The summary statistics of the clinical data is available in the main text or the supplementary materials.
