# Supplementary material for: Systematic identification of ACE2 expression modulators reveals cardiomyopathy as a risk factor for mortality in COVID-19 patients
Source: Genome Biol. 2022 Jan 10;23:15. doi: 10.1186/s13059-021-02589-4 (PMC8743438; doi:10.1186/s13059-021-02589-4)
Supplement: Supplementary file 1 — Additional file 1: Supplemental figures and tables. This file including figure S1, figure S2, table S8 and table S9. [file 13059_2021_2589_MOESM1_ESM.pdf]

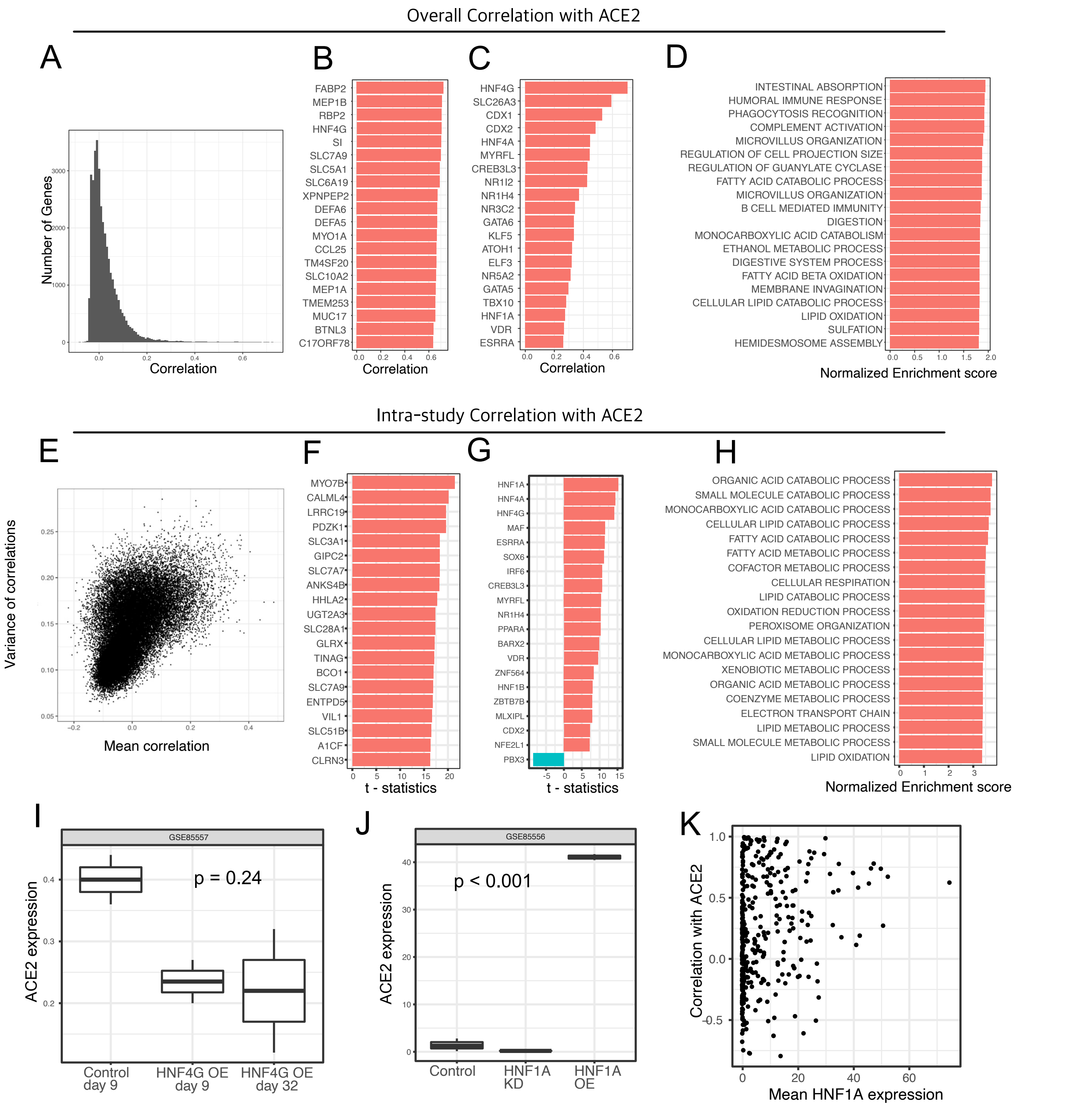

Figure S1: RNA-seq analysis reveals complex transcriptional networks of ACE2. We calculated the pairwise Pearson correlations between ACE2 and other genes using all data from 286650 RNA-seq samples (A-D). (A) Histogram of the pairwise correlations. (B) Top 30 genes with the highest correlation with ACE2. (C) Top 30 transcription factors with the highest correlation with ACE2. (D) Top 30 pathways correlated with ACE2 expression. We calculated the pairwise Pearson correlations between ACE2 and other genes using samples in individual datasets (E-H). (E) Scatter plot shows the mean and variance of the correlation between ACE2 and other genes across different datasets. (F) Top 30 genes with the highest association (measured by the t statistics from mix-effect models that control for study differences) with ACE2. (G) Top 30 transcription factors with the highest association (measured by the t statistics from mix-effect models that control for study differences) with ACE2. (H) Top 30 pathways with the highest associations with ACE2. (I) Box plot showing the expression of ACE2 in LNCaP/AR cells with or without HNF4G over expression. (I) Box plot showing the expression of ACE2 in LNCaP/AR cells with or without HNF4A over expression or knock down. (K) scatter plot shows the relationship between HNF4G expression level and its correlation with ACE2.

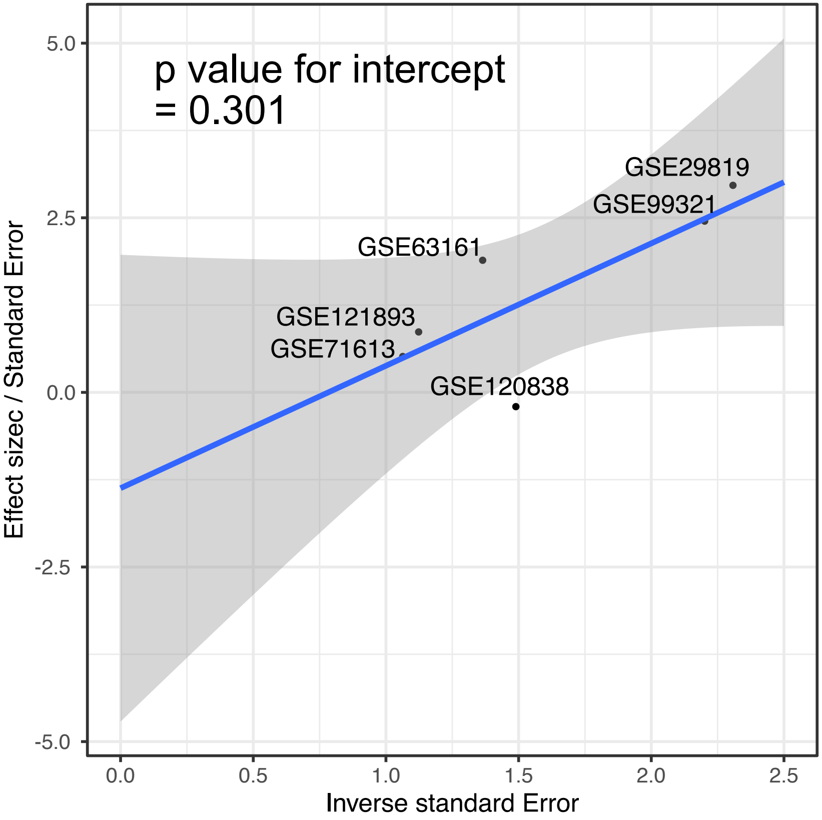

Figure S2: Test the publication bias in the cardiomyopathy-related datasets using Egger regression.

	pre-existing cardiomyopathy (N=43)	Other cardiovascular diseases (N=344)	Overall (N=387)
**age**			
Mean (SD)	58.6 (17.9)	57.9 (19.7)	58.0 (19.5)
Median [Min, Max]	60.0 [18.0, 89.0]	60.5 [0, 100]	60.0 [0, 100]
**gender**			
Female	10 (23.3%)	95 (27.6%)	105 (27.1%)
Male	33 (76.7%)	249 (72.4%)	282 (72.9%)
**race**			
Asian	12 (27.9%)	77 (22.4%)	89 (23.0%)
Black or African American	5 (11.6%)	33 (9.6%)	38 (9.8%)
MultiRace	1 (2.3%)	9 (2.6%)	10 (2.6%)
Native Hawaiian or Other Pacific Islander	3 (7.0%)	13 (3.8%)	16 (4.1%)
Unknown	9 (20.9%)	74 (21.5%)	83 (21.4%)
White	13 (30.2%)	138 (40.1%)	151 (39.0%)
**hyperlipidemia**			
Yes	27 (62.8%)	195 (56.7%)	222 (57.4%)
No	16 (37.2%)	149 (43.3%)	165 (42.6%)
**diabetes**			
Yes	27 (62.8%)	184 (53.5%)	211 (54.5%)
No	16 (37.2%)	160 (46.5%)	176 (45.5%)
**cancer**			
Yes	10 (23.3%)	81 (23.5%)	91 (23.5%)
No	33 (76.7%)	263 (76.5%)	296 (76.5%)
**ventilator use**			
Yes	24 (55.8%)	90 (26.2%)	114 (29.5%)
No	19 (44.2%)	254 (73.8%)	273 (70.5%)
**respiratory failure**			
Yes	12 (27.9%)	62 (18.0%)	74 (19.1%)
No	31 (72.1%)	282 (82.0%)	313 (80.9%)
**chest pain**			
Yes	27 (62.8%)	125 (36.3%)	152 (39.3%)
No	16 (37.2%)	219 (63.7%)	235 (60.7%)
**death**			
Yes	3 (7.0%)	4 (1.2%)	7 (1.8%)
No	40 (93.0%)	340 (98.8%)	380 (98.2%)

Table S8: Demographic and clinical information of COVID-19 patients with cardiomyopathy and a propensity score matched cohort of COVID-19 patients with other cardiovascular diseases

	Cardiomyopathy (N=2250)	Other cardiovascular diseases (N=18000)	Overall (N=20250)
**age**			
Mean (SD)	45.9 (23.3)	46.4 (24.1)	46.3 (24.0)
Median [Min, Max]	50.0 [0, 90.0]	51.0 [0, 91.0]	51.0 [0, 91.0]
**gender**			
FEMALE	897 (39.9%)	7238 (40.2%)	8135 (40.2%)
MALE	1352 (60.1%)	10758 (59.8%)	12110 (59.8%)
UNKNOWN	1 (0.0%)	4 (0.0%)	5 (0.0%)
**race**			
American Indian or Alaska Native	9 (0.4%)	73 (0.4%)	82 (0.4%)
Asian	200 (8.9%)	1526 (8.5%)	1726 (8.5%)
Black or African American	190 (8.4%)	1539 (8.6%)	1729 (8.5%)
MultiRace	122 (5.4%)	1018 (5.7%)	1140 (5.6%)
Native Hawaiian or Other Pacific Islander	27 (1.2%)	195 (1.1%)	222 (1.1%)
Other Race	382 (17.0%)	3172 (17.6%)	3554 (17.6%)
Unknown	335 (14.9%)	2449 (13.6%)	2784 (13.7%)
White	985 (43.8%)	8028 (44.6%)	9013 (44.5%)
**hyperlipidemia**			
Yes	368 (16.4%)	2945 (16.4%)	3313 (16.4%)
No	1882 (83.6%)	15055 (83.6%)	16937 (83.6%)
**diabetes**			
Yes	370 (16.4%)	2874 (16.0%)	3244 (16.0%)
No	1880 (83.6%)	15126 (84.0%)	17006 (84.0%)
**cancer**			
Yes	306 (13.6%)	2430 (13.5%)	2736 (13.5%)
No	1944 (86.4%)	15570 (86.5%)	17514 (86.5%)
**death**			
Yes	123 (5.5%)	807 (4.5%)	930 (4.6%)
No	2127 (94.5%)	17193 (95.5%)	19320 (95.4%)

Table S9: Demographic and clinical information of Non-COVID-19 patients with cardiomyopathy and a propensity score matched cohort of patients with other cardiovascular diseases
