# Supplementary material for: Systematic identification of ACE2 expression modulators reveals cardiomyopathy as a risk factor for mortality in COVID-19 patients
Source: Genome Biol. 2022 Jan 10;23:15. doi: 10.1186/s13059-021-02589-4 (PMC8743438; doi:10.1186/s13059-021-02589-4)
Supplement: Supplementary file 9 — Additional file 9:. Review history. [file 13059_2021_2589_MOESM9_ESM.docx]

**1^st^ round**

**Reviewer 1**

Kaur et al. perform a systematic investigation of ACE2 expression profile in various biological settings (e.g., diseases, drugs). They report a potential causal pathway in which cardiomyopathy modulates ACE2 expressions and leads to increased mortality of COVID-19. The amount of computational work undertaken by the authors is impressive. However, I am concerned with the authors' interpretations of their computational results, especially for the causality claims. I hope the following comments help the authors revise the manuscript.

1. I find quite a few causal claims, despite striking, are quite loosely interpreted. Almost all evidence presented in the paper is correlations by nature. There is a high bar to interpret such evidence as to causation. Taking the GENEVA results as an example, the authors claim to identify relevant diseases *modulating* the expression of ACE2. However, I can not tell how the selected datasets lead to such a conclusion (From the description on P 19, the datasets look observational. The subsequent regression and sub-type analyses also do not help establish "modulating" effects.). Similarly, The EHR analysis identifies correlations between COVID-19 mortality and cardiomyopathy. This correlation is expected if the hypothesized causal relationship is true. However, it does not strengthen the causal claims. The authors should make this point clear. My general suggestion is that the authors should be more careful about the causality claims. This can be achieved by discussing the experiment designs of relevant datasets (e.g., intervention vs. observation). If the dataset is indeed observational, some causal inference techniques should be applied, and potential unknown confounding should be discussed and acknowledged.

2. I find the joint analysis of ACE2 expression across multiple datasets oversimplified. The procedure described in the last paragraph seems overly ad-hoc. What is the rationale for weighing each estimate by the sample size, given the variance is available from each dataset? (Also, do they mean the inverse of the sample size?) I also do not understand the necessity of introducing the "standardized correlation coefficient," a concept that does not seem well-motivated.

3. I am a bit confused about the claim that GENEVA can identify datasets "in which ACE2 is regulated by experimental conditions rather than randomness or unexplained factors". My understanding is that the proposed procedure aims to identify correlations consistently presented across datasets. If so, I am not sure the proposed permutation procedure is appropriate for this purpose. The use of p-values and the permutation test is problematic in multiple ways. Most importantly, how is a rejection of the null hypothesis (asserting no association in all datasets) leading to a consistency claim of non-zero correlation?

4. I think the claim of "unbiased meta-analysis" needs to be better explained/examined. The claim is seemingly related to the publication bias/winner's curse. If this is the case, the authors should show evidence from relevant diagnosis tests, e.g., funnel plots, Egger regression. Note that taking a meta-analysis by including all available studies does not automatically eliminate such bias. There is rich literature on the topic. Additionally, what is a "meta-analysis using a mixed-effect"? Do they mean random-effect meta-analysis?

**Reviewer 2**

This study promises to offer an important resource to the scientific community. The analysis of ACE2 is timely, and the results will be of interest both from a basic science and translational perspective. The code is available and a website of results has been developed. However, there are major methodological and statistical gaps in the study, as currently presented. The following must be addressed to improve interpretability, utility, and reproducibility.

1. Pages 5-6: Some of the gene expression datasets included, though heterogeneous, may not be independent. Was the correlation between ACE2 and the remaining genes calculated without taking this into account? How was the correlation threshold of 0.5 chosen?

2. Page 6: How were the "highly expressed genes" chosen? What definition was used? How was the similarity in expression pattern between these genes and ACE2 assessed? Claims were made here without sufficient justification.

3. Page 7 line 16-17: Shouldn't "HNF1G" be "HNF4G"?

4. Page 18: How was the variance threshold of 10 chosen? Some of the choices, without any justication, seem arbitrary.

5. Page 39: Top 30 transcription factors, top 30 pathways, top 30 genes with highest standardized correlation with ACE2, top 30 pathways with highest standarized correlation with ACE2: How was the threshold (30) chosen for each of these? Please provide justification. Many of the choices seem arbitrary rather than statistically motivated.

6. Methodologically, why did the authors choose not to use a standardized measure of dispersion (e.g., coefficient of variation) rather than just variance in the definition of GENEVA score?

7. Page 6: How was the weighing by sample sizes done? Clearly, the studies have high variation in sample size. It's important to clarify explicitly how this can impact the conclusions that can be drawn.

**Authors’ response**

We thank both reviewers for the suggestions and comments. We have updated our manuscript based on the comments (highlighted in blue in the manuscript file). We have also performed additional analysis and have included new figures and tables (Figure S2, Table S1-7) in the updated manuscript. Please see our point-by-point responses below.

Reviewer #1: Kaur et al. perform a systematic investigation of ACE2 expression profile in various biological settings (e.g., diseases, drugs). They report a potential causal pathway in which cardiomyopathy modulates ACE2 expressions and leads to increased mortality of COVID-19.  The amount of computational work undertaken by the authors is impressive. However, I am concerned with the authors' interpretations of their computational results, especially for the causality claims. I hope the following comments help the authors revise the manuscript.

1. I find quite a few causal claims, despite striking, are quite loosely interpreted. Almost all evidence presented in the paper is correlations by nature. There is a high bar to interpret such evidence as to causation. Taking the GENEVA results as an example, the authors claim to identify relevant diseases *modulating* the expression of ACE2. However, I can not tell how the selected datasets lead to such a conclusion (From the description on P 19, the datasets look observational. The subsequent regression and sub-type analyses also do not help establish "modulating" effects.). Similarly, The EHR analysis identifies correlations between COVID-19 mortality and cardiomyopathy. This correlation is expected if the hypothesized causal relationship is true. However,  it does not strengthen the causal claims. The authors should make this point clear.  My general suggestion is that the authors should be more careful about the causality claims. This can be achieved by discussing the experiment designs of relevant datasets (e.g., intervention vs. observation).  If the dataset is indeed observational, some causal inference techniques should be applied, and potential unknown confounding should be discussed and acknowledged.

Response to the reviewer: we apologize for using the term “modulating” loosely in the manuscript. The goal of this study is to identify conditions that are associated with ACE2 expression. While causality cannot be established in many cases, the associations identified in this study could help us find potential risk factors for severe COVID-19 cases.

In the updated manuscript, we carefully examined the experimental design of the four highlighted conditions (cardiomyopathy, HNF1A overexpression, drug treatments with RAD140 and Itraconazole). Among them, causal relationships can be established for three conditions (HNF1A overexpression, and drug treatments with RAD140 and Itraconazole), since ACE2 expression change was observed after the experimental manipulation in controlled experiments. For example, we observe a higher ACE2 expression in cell lines treated with Itraconazole than the controls, thus establishing a causal relationship between Itraconazole treatment and ACE2 up-regulation.

The cardiomyopathy studies are observational in nature and do not prove causal relationships between cardiomyopathy and ACE2 expression. We have revised our language in the manuscript to remove any causal claims and acknowledged the lack of causal evidence in the discussion section. While not causal, the observed high ACE2 expression suggests that cardiomyopathy patients are a high-risk group during the COVID-19 pandemic, which was later strengthened by our medical record analysis.

2. I find the joint analysis of ACE2 expression across multiple datasets oversimplified. The procedure described in the last paragraph seems overly ad-hoc. What is the rationale for weighing each estimate by the sample size,  given the variance is available from each dataset? (Also, do they mean the inverse of the sample size?) I also do not understand the necessity of introducing the "standardized correlation coefficient," a concept that does not seem well-motivated.

Response to the reviewer. we have updated our methodology for analyzing ACE2 correlations across datasets. Instead of using an ad-hoc procedure, we adopted a mixed effect model [Gene ~ ACE2 (fixed effect) + dataset ID (random effect for intercept and slope)] for assessing the association between ACE2 and other genes, while including the dataset ID as a random effect for both intercept and slope. The random effect on the slope allows us to capture the variability of the co-expression between ACE2 and other genes across studies. The t statistics of the fixed effect allow us to test the overall significance of the association between ACE2 and other genes. We have updated the related result in Figure S1 and added new supplemental tables (Table S5-7) to report the results.

3. I am a bit confused about the claim that GENEVA can identify datasets "in which ACE2 is regulated by experimental conditions rather than randomness or unexplained factors". My understanding is that the proposed procedure aims to identify correlations consistently presented across datasets. If so, I am not sure the proposed permutation procedure is appropriate for this purpose. The use of p-values and the permutation test is problematic in multiple ways. Most importantly, how is a rejection of the null hypothesis (asserting no association in all datasets) leading to a consistency claim of non-zero correlation?

Response to the reviewer. We would like to clarify that the goal of GENEVA is to identify individual datasets in which ACE2 expressions are associated with experimental conditions, rather than identifying consistent correlations across datasets. For example, figure 2 highlighted three separate datasets in which ACE2 expressions are associated with cardiomyopathy, RAD140 treatment and Itraconazole treatment. Because these datasets studied unrelated conditions, GENEVA did not attempt to find results that are consistent across datasets.

The GENEVA score is a statistics that reflects the association between ACE2 expression and the embedding of sample labels. High GENEVA score indicates high association between ACE2 and the experimental groups. However, high GENEVA score could rise purely by chance. Thus, we designed a permutation method to test the significance of the GENEVA score. We shuffle the sample labels within each dataset to break down the association between the ACE2 and sample groups. The GENEVA scores from the shuffled datasets create a null distribution, which can be used to test the significance of Geneva score from un-permutated datasets.

Please note that the correlations between ACE2 and other genes were reported in Figure S1 and were conducted separately from GENEVA. In the new manuscript, we have used mixed effect models to identify consistent correlations between ACE2 and other genes across datasets.

4. I think the claim of "unbiased meta-analysis" needs to be better explained/examined. The claim is seemingly related to the publication bias/winner's curse. If this is the case, the authors should show evidence from relevant diagnosis tests, e.g., funnel plots, Egger regression. Note that taking a meta-analysis by including all available studies does not automatically eliminate such bias. There is rich literature on the topic.

Response to the reviewer: We thank the reviewer for bringing up the topic of publication bias. It is true that such bias exists extensively. In our study, we are repurposing the existing RNA-seq dataset to study the expression of ACE2. Since ACE2 expression is not of interest to the original authors and the publication of the original RNA-seq study is not likely to be affected by the effect size of one gene, our meta-analysis is unlikely to be affected by publication bias.

In the revised manuscript, we used the Egger regression to further examine publication bias in the cardiomyopathy datasets. We did not observe obvious publication bias. The new result has been added as supplemental figure 2 in the revised manuscript.

Additionally, what is a "meta-analysis using a mixed-effect"? Do they mean random-effect meta-analysis?

Response to the reviewer: In our analysis, we used a mixed-effect model (expression ~ cardiomyopathy (fixed effect) + dataset ID (random effect)) to test the association between ACE2 expression and the cardiomyopathy across studies. We realized that the term “meta-analysis” may be misleading, since it often refers to the practice of combining summary statistics rather than sample-level data across studies. Our analysis uses a mixed effect model to analyze the sample-level expression data from the 7 datasets. In the updated manuscript, we used the term 'joint analysis' to avoid confusion.

Reviewer #2: This study promises to offer an important resource to the scientific community. The analysis of ACE2 is timely, and the results will be of interest both from a basic science and translational perspective. The code is available and a website of results has been developed. However, there are major methodological and statistical gaps in the study, as currently presented. The following must be addressed to improve interpretability, utility, and reproducibility.

1. Pages 5-6: Some of the gene expression datasets included, though heterogeneous, may not be independent. Was the correlation between ACE2 and the remaining genes calculated without taking this into account? How was the correlation threshold of 0.5 chosen?

Response to the reviewer: we thank the reviewer for the comment on the dependency between the datasets. The gene expression datasets are independent in the sense that each dataset is generated from a separated experiment. We noticed that an RNA-sequencing sample could belong to more than one datasets in the GEO database, suggesting that these datasets are generated from related experiments. To ensure the independency between datasets, our analysis pipeline excluded the datasets that share samples with other datasets. We have emphasized these exclusion criteria in the method section of the updated manuscript.

We realized that a correlation threshold of 0.5 is arbitrary. Please note that the threshold was only used to describe the long-tailed distribution (shown in figure S1A) and did not affect the downstream analysis and interpretation. In the new manuscript, we have updated our description to avoid using 0.5 as a threshold.

2. Page 6: How were the "highly expressed genes" chosen? What definition was used? How was the similarity in expression pattern between these genes and ACE2 assessed? Claims were made here without sufficient justification.

Response to the reviewer: we have updated our analysis to 1) avoid using hand-selected genes and 2) qualify the similarity of expression pattern between ACE2 and other genes across tissue types. We calculated the correlation between ACE2 and all other genes using tissue-specific data from GTEX and have reported the results in Table S4.

3. Page 7 line 16-17:  Shouldn't "HNF1G" be "HNF4G"?

Response to the reviewer: we thank the reviewer for the correction. Indeed, it should be HNF4G. We have made the correction in the revised manuscript.

4. Page 18: How was the variance threshold of 10 chosen? Some of the choices, without any justication, seem arbitrary.

Response to the reviewer: we have removed the threshold of 10 and used all datasets to assess the correlation between ACE2 and other genes. Please see the updated method section, Figure S1 F-H, and table S5-7.

5. Page 39: Top 30 transcription factors, top 30 pathways, top 30 genes with highest standardized correlation with ACE2, top 30 pathways with highest standarized correlation with ACE2:  How was the threshold (30) chosen for each of these?  Please provide justification. Many of the choices seem arbitrary rather than statistically motivated.

Response to the reviewer: as we have mentioned in the manuscript, because of the large sample size in our study, we observe significant correlations between ACE2 and most genes or pathways, even after the most conservative multiple testing adjustment (Bonferroni correction). Therefore, selecting genes based on standard p values is not practical in our case, not to mention that the well-accepted 0.05 threshold for p values is an arbitrary choice itself based on historical traditions.

In the new manuscript, we have clarified that the top 30 genes or pathways are chosen for visualization purposes due to space constrains. We have also added 7 new supplemental tables (table S1-7) to list all the associations between ACE2 and genes or pathways, allowing the researchers to examine the result beyond the top 30 results.

6. Methodologically, why did the authors choose not to use a standardized measure of dispersion (e.g., coefficient of variation) rather than just variance in the definition of GENEVA score?

Response to the reviewer: when calculating the GENEVA score, we used the variance rather than a standardized dispersion. This choice is made on purpose to identify studies that show large up-regulation/down-regulation of ACE2. Experimental studies have demonstrated the cell entry of SARS-COV-2 is directly associated with the expression level of ACE2 in the cell, as shown by the observation that the gradient of ACE2 expression mirrors the profile of permissiveness to SARS-CoV-2 (Hou et al., 2020, Cell). Therefore, we reason that the datasets with large variations of ACE2 expression (e.g. ACE2 up-regulation from 10 TPM to 30 TPM) are more biologically relevant in the context of SARS-Cov-2 infection than datasets with smaller ACE2 variances (e.g. ACE2 up-regulation from 0.1 TPM to 0.3 TPM), even though the two datasets have similar coefficient of variation.

7. Page 6: How was the weighing by sample sizes done? Clearly, the studies have high variation in sample size. It's important to clarify explicitly how this can impact the conclusions that can be drawn.

Response to the reviewer: we thank the reviewer for the comments on the "weighing by sample sizes” procedure. Based on the comments from both reviewers, we have updated our methodology for analyzing ACE2 correlations across datasets. Instead of an ad-hoc “weighing by sample sizes” procedure, we adopted a mixed effect model [Gene ~ ACE2 (fixed effect) + dataset ID (random effect for intercept and slope)] for assessing the association between ACE2 and other genes, while including the dataset ID as a random effect for both intercept and slope. The random effect on the slope allows us to capture the variability of the associations between ACE2 and other genes across studies, and the fixed effect allows us to test the overall significance of the association between ACE2 and other genes. We have updated the method section and the related results in Figure S1 and Supplemental Table 5-7.

**2^nd^ round**

**Reviewer 2**

The authors have addressed my concerns.
